# Methods to appraise available evidence and adequacy of data from a systematic literature review to conduct a robust network meta-analysis of treatment options for patients with hospital-acquired or ventilator-associated bacterial pneumonia

**DOI:** 10.1371/journal.pone.0279844

**Published:** 2023-01-04

**Authors:** Laura Puzniak, Ryan Dillon, Thomas Lodise

**Affiliations:** 1 Merck & Co., Inc., Rahway, New Jersey, United States of America; 2 Department of Pharmacy Practice, Albany College of Pharmacy and Health Sciences, Albany, New York, United States of America; University of South Carolina College of Pharmacy, UNITED STATES

## Abstract

We aimed to determine if available evidence from a previously conducted systematic literature review was sufficient to conduct a robust network meta-analysis (NMA) using the International Society for Pharmacoeconomics and Outcomes Research Good Practice Task Force NMA study questionnaire to evaluate suitability, relevance, and credibility of available randomized-controlled trials (RCT) of antibacterial therapies for treatment of patients with hospital-acquired or ventilator-associated bacterial pneumonia (HABP/VABP). We assessed feasibility and reliability of an NMA for a connected network of RCTs, and then relevance and credibility of the connected network for informing decision-making. This previously conducted systematic literature review using Cochrane dual-reviewer methodology, Preferred Reporting Items for Systematic Reviews and Meta-Analyses guidelines, and PICOTS (population, interventions, comparators, outcomes, timing, and setting) criteria identified 25 citations between 2001 and 2018; 18 were unique RCTs. Trial design characteristics, outcome definitions, assessment time points, and analyses populations varied across studies. Using “clinical response,” an efficacy end point to health technology assessment agencies, we assessed potential network credibility, which collapsed from the overall data set to four studies and five interventions. This did not include closed loop(s) needed to assess consistency. Of the studies reporting clinical response, >70% of patients were ventilated at baseline with mean Acute Physiologic Assessment and Chronic Health Evaluation II scores from 14.7 to 17.5. *Pseudomonas aeruginosa* (range, 18.4–64.1%) and *Klebsiella* spp. (range, 1.6–49%) were the most common causative pathogens. We identified relevant RCTs for most standard-of-care agents approved for HABP/VABP, which provided a comprehensive evidence base. In summary, our appraisal of available evidence for the clinical response outcome among adult patients with HABP/VABP does not support the conduct of a scientifically robust and clinically meaningful NMA. Although this data is vital to registration, there are significant limitations in these trials for health technology assessments, payor decisions, guidelines, and protocol decisions.

## Introduction

In recent years, several antibacterial agents have been approved for the treatment of adult patients with hospital-acquired/ventilator-associated bacterial pneumonia (HABP/VABP) caused by gram-negative pathogens [1]. Like other therapeutic domains, approval of these agents require many countries with national health care systems to evaluate safety, efficacy, and value to obtain formulary status and reimbursement. Currently, randomized-controlled trials (RCT) are considered the gold standard in the evaluation of health care policy decision-making in many countries and are considered the best type of study to determine whether there is a causal relationship between the intervention and the effect [2]. Ideally, comprehensive health technology assessments (HTA) are based on the results from well-designed and conducted RCTs that simultaneously compare all interventions of interest across all relevant outcomes. However, such RCTs are rarely available, especially for infectious conditions like HABP/VABP.

In the absence of comprehensive RCTs that include all relevant comparators, evidence synthesis methods such as meta-analysis and indirect treatment comparisons (ITC) are being increasingly used by policy decision-makers and reimbursement authorities for judiciously selecting the optimal treatment(s) when direct treatment comparisons between competing inventions are lacking [3–5]. The most common ITC method is a standard network meta-analysis (NMA), which provides estimates of relative treatment effects across multiple treatments based on direct treatment comparisons and/or ITCs in a connected network of RCTs that assess common outcomes or have common comparator arms [6, 7]. If direct treatment comparisons or NMAs are not possible, ITC approaches may be employed and include naïve and matching-adjusted indirect comparisons [8]. In contrast to NMA, “unanchored” naïve or matching-adjusted indirect comparisons do not rely on an RCT network that includes a common comparator arm; however, it has been noted that this approach may introduce bias in the interpretation of the results [8, 9].

Although there is increased use of NMA and other ITC methods to inform health care decisions in infectious diseases [10], there are challenges in appropriately applying and interpreting their results for decision-making. Similar to meta-analyses, there are critical feasibility and validity considerations when designing and conducting NMAs, as they are highly vulnerable to random and systematic biases [11]. It is of paramount importance to assess potential internal and external validity threats when making health care technology policy decisions from NMA and other ITC methods for conditions like HABP/VABP. Unlike other therapeutic domains that rely on superiority demonstrated in RCTs to define best therapies and practices [12], ethical concerns prevent placebo-controlled superiority trials for HABP/VABP owing to high mortality rates and the availability of effective antibacterial therapies [13]. Both the United States Food and Drug Administration and European Medicines Agency have predominantly based approvals for new antibacterial agents on multicenter, double-blind, randomized, noninferiority phase 3 RCTs, and have established guidelines to support use of the noninferiority trial design, using either a placebo or investigational drug added to a standard-of-care HABP/VABP treatment as the comparator [14–17]. To ensure clinical equipoise, one of the additional requirements for noninferiority trials for HABP/VABP is the exclusion of patients with suspected or documented infections that are resistant to the active comparator and this may further exacerbate external validity concerns (ie, context) [13]. Given the increased consideration for NMA in informing health care policy in infectious diseases, we sought to determine if the available RCT data for interventions treating HABP/VABP were adequately feasible and reliable to support the subsequent conduct of a valid NMA, which in turn could be used to inform health care decision-making. We conducted a systematic literature review (SLR) and examined the resulting RCT data for suitability, relevance, and credibility of a potential NMA within the antibacterial therapeutic space for the indication of HABP/VABP.

## Methods

### Identification of the HABP/VABP evidence base

An SLR was conducted to identify relevant studies for consideration in an NMA for treatment of HABP/VABP. This SLR was conducted using a standardized approach after Cochrane dual-reviewer methodology (S1 File. Study methodology) [18]. The SLR protocol followed the Preferred Reporting Items for Systematic Reviews and Meta-Analyses (PRISMA) guidelines [19] and used the PICOTS (population, interventions, comparators, outcomes, timing, and setting) criteria (S1 Table. SLR PICOTS criteria for study eligibility) to evaluate all publications. The databases that were searched included MEDLINE®, EMBASE®, and the Cochrane Central Register of Controlled Trials via the OVID® platform, with publications between 2000 and 2019. The search terms were related to the brand and generic names of antibacterial agents of interest and the HABP/VABP disease area and included terms and study design filters recommended by the Scottish Intercollegiate Guidelines Network [20] for identifying clinical trials in MEDLINE and EMBASE. Detailed processes and methodologies used to conduct this SLR are described in S2 Table. Detailed search strategy.

### Network meta-analysis

To determine the feasibility and reliability of a potential NMA within the antibacterial therapeutic space for the indication of HABP/VABP, we applied the International Society for Pharmacoeconomics and Outcomes Research (ISPOR) Good Practice Task Force report, “Indirect Treatment Comparison/Network Meta-analysis Study Questionnaire to Assess Study Relevance and Credibility to Inform Health Care Decision-Making” to the results of the SLR [21]. To inform this appraisal, we used the results from the SLR and corresponding data extraction tables to evaluate point-by-point the two constructs from the questionnaire for a valid NMA, relevance, and credibility. This review provides a point-by-point illustration of important considerations related to the potential use of NMAs for valid comparison and decision-making.

First, we assessed the potential for a connected network of RCTs based on the available data. Second, we assumed it was possible that a network could be created and assessed the overall relevance of the feasible connected network. Third, credibility of the theoretical connected network was assessed for informing decision-making. Using the ISPOR Good Practice Task Force NMA study questionnaire, the domains of analysis, reporting quality and transparency, interpretation.

### Assessment of relevance

“Relevance” addresses the degree to which the results of the NMA analysis aligns with the perspective of the decision-maker and focuses on the applicability and credibility of the study population, intervention, and outcomes [21]. To determine “relevance,” our review adopted the perspective of an HTA decision-maker who considers clinical outcome data, (ie, efficacy and safety) to inform economic models and/or estimates of comparative clinical effectiveness. We assessed if any potential connected network of RCTs of interventions for HABP/VABP was possible to evaluate studies identified from the SLR using the following four questions described in the ISPOR questionnaire: 1) Is the study population relevant? 2) Are any relevant interventions missing? 3) Are any relevant outcomes missing? and 4) Is the context applicable? For our relevance review, we evaluated the comparability and clinical applicability of the HABP/VABP populations, treatments, and outcomes across the studies included in the NMA. For context, we evaluated if the patients in the RCT that were included in the NMA reflected the setting and circumstances of HABP/VABP that were of greatest interest to HTA decision-makers.

### Assessment of credibility

The domain of “credibility” was determined by review of data in the SLR from an HTA viewpoint using the following questions: 1) Did the SLR a priori eligibility criteria admit all relevant studies and were sufficient sources searched? 2) Can treatments in the identified studies be compared directly or indirectly? 3) Were the studies of good quality, including reporting bias, differences in effect modifiers, and imbalance of effect modifiers [21]? We focused the “credibility” assessment on those studies able to create a connected network of all important interventions and on the presence for systematic differences in treatment effect modifiers (ie, baseline patient or study characteristics that have an impact on the treatment effects) across the different treatment comparisons in the network, with a particular concentration on clinical response.

## Results

### Systematic literature review

**Fig 1:**
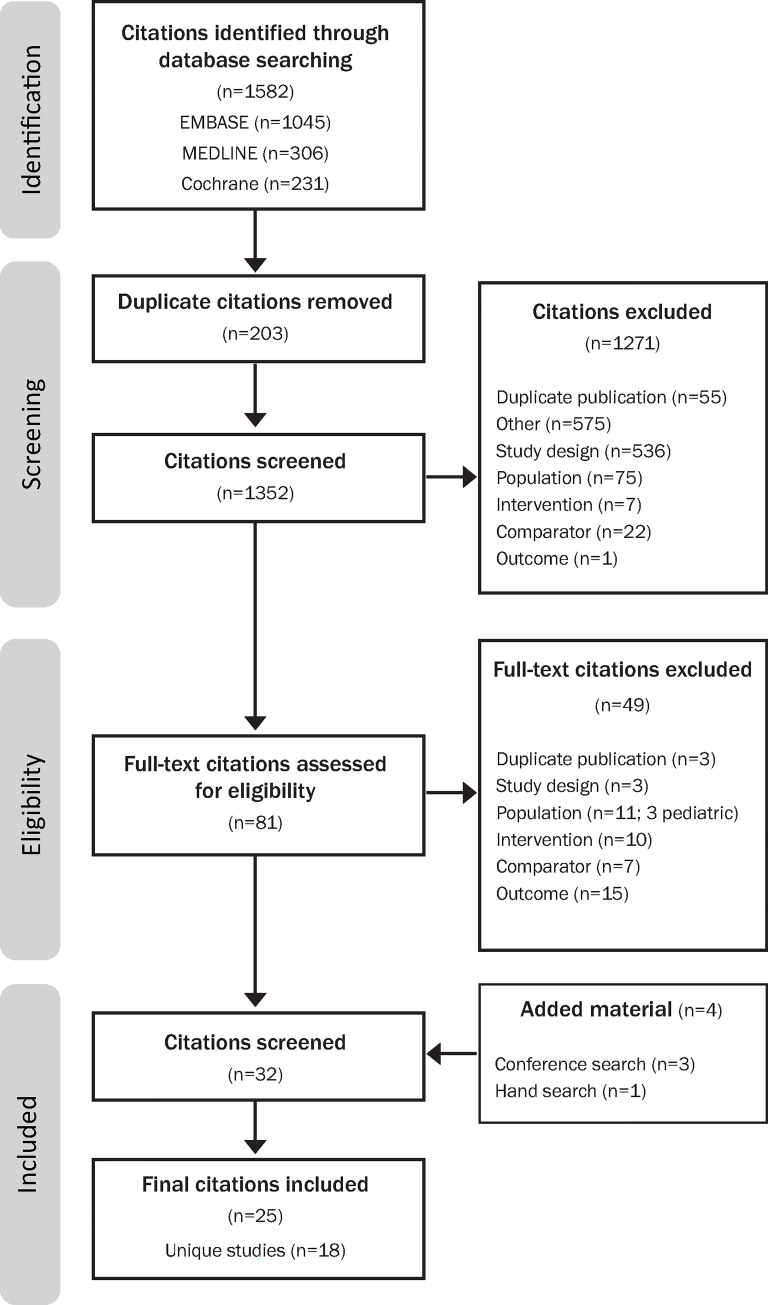
PRISMA diagram.

The previously conducted SLR identified 25 citations [22–50] between 2001 and 2018, corresponding to 18 unique RCTs [22–30, 32–40, 42, 43] after accounting for those with multiple publications (Fig 1). Trial design characteristics are summarized in Table 1. The RCTs were conducted in global trials (n = 3), regional locations (n = 10), and some did not report locations (n = 5). Study designs included open-label (n = 10) and blinded (n = 7); the study design was not reported for one study. Baseline stratification factors were reported in 33% (6/18) of the studies. Of the included studies, each reported two treatment arms, with the exception of Damas et al. [27], which randomized patients into three treatment arms. All of the studies admitted patients with either HABP and/or VABP; and one study (ASPECT-NP [31]) enrolled ventilated patients exclusively (patients with ventilated HABP and VABP). Half of the studies had exclusion criteria pertaining to prior antibacterial therapy and 61% (11/18) of the studies exclusion criteria addressed renal status.

**Table 1:** Trial design characteristics.

Trial ID	Study start and end date	Masking	Study region	Baseline stratification factors	Exclusion criteria			
					Prior antibacterial therapy	Renal status	Expected survival	APACHE II scores
**Studies reporting clinical response (n = 4) within ASPECT-NP–connected network**								
Alvarez-Lerma 2001 phase NR [23] N = 140	–	Open-label	Spain	NR	Antibacterial treatment active against the pathogens responsible for the pneumonia in the 72 hours before the initial administration of the study medication	Renal insufficiency	<1 month	NR
Alvarez-Lerma 2001 phase 3 [24] N = 124	–	Open-label	Spain	NR	Antibiotic treatment within 72 hours before inclusion in the study that were active against causative pathogens of pneumonia	Renal failure (serum creatinine concentration >3.5 mg/dL or creatinine clearance <20 mL/min)	<1 month	NR
Kollef 2019 ASPECT-NP phase 3 [31] N = 726	Sep 2014–Jun 2018	Quadruple-blind	NR	NP diagnosis (VABP and vHABP) and age (≥65 and <65 years)	Prior nonstudy systemic or inhaled antibacterial therapy with activity against gram-negative pathogens that cause ventilated NP, for >24 hours in the 72 hours before first dose of study drug	End-stage renal disease or requirement for dialysis	<72 hours	NR
Torres 2018 REPROVE phase 3 [35] N = 726	Apr 2013–Dec 2015	Double-blind	Argentina, Brazil, Bulgaria, China, Czechia, France, Hungary, India, Italy, Japan, Korea, Latvia, Mexico, Peru, Philippines, Poland, Romania, Russian Federation, Slovenia, South Africa, Spain, Taiwan, Turkey, Ukraine, United Kingdom, Vietnam	Infection type (ie, ventilator-associated or nonventilator-associated) and geographical region (Western Europe, Eastern Europe, China, rest of the world)	NR	Patients receiving hemodialysis or peritoneal dialysis	NR	NR
**Remaining studies reporting HABP/VABP meeting SLR eligibility criteria**								
Ahmed 2007 phase NR [22] N = 93	Apr 2004–Mar 2005	NR	India	NR	NR	Acute renal failure or chronic renal failure	NR	NR
Chastre 2008 phase 3 [25] N = 248	NR	Open-label	North America, Europe, Australia	Duration of mechanical ventilation (<5 vs >5 days), severity of illness (APACHE II score <15 vs >15), and geographic region	Systemic antibiotic therapy for 24 hours in the 48 hours before randomization	End-stage renal failure (including any form of dialysis)	Considered unlikely to survive until the final study visit	<8 or >29
Chaudhary 2008 phase NR [26] N = 200	Sep 2005–Mar 2006	Open-label	India	NR	NR	Renal insufficiency	NR	NR
Damas 2006 phase NR [27] N = 59	Apr 2002–Dec 2003	Open-label	Belgium	NR	NR	NR	NR	NR
Heyland 2008 phase NR [28] N = 739	May 2000–Feb. 2005	Open-label	United States, Canada	Site and illness severity (APACHE II score ≤24 or >24 in the 24-hour period before enrollment)	NR	NR	Die within 24 hours	NR
Joshi 2006 phase NR [29] N = 437	Apr 1997–Dec 2001	Double-blind	United States, Canada	NR	NR	Renal insufficiency	NR	NR
NCT00515034 phase 2 [38] N = 64	Oct 2007–Nov 2008	Open-label	NR	NR	NR	Renal insufficiency	NR	NR
NCT00589693 phase 3 [37] N = 227	NR	Double-blind	NR	NR	Received antibiotics for this episode of ventilator-associated pneumonia for >24 hours before study medication administration	NR	NR	≤8 or ≥35
RESTORE-IMI 1 phase 3 [32] N = 31	Aug 2015–Sep 2017	Double-blind	NR	Infection type	Received treatment with any form of systemic colistin for >24 hours within 72 hours before initiation of study drug (for groups 1 and 2 only)	Currently undergoing hemodialysis or peritoneal dialysis	NR	>30
RESTORE-IMI 2 phase 3 [51] N = 531	Aug 2015–Sep. 2017	Double-blind	United States, Argentina, Australia, Brazil, Bulgaria, Canada, Colombia, Croatia, Estonia, France, Guatemala, Italy, Japan, Korea, Latvia, Lithuania, Mexico, Norway, Peru, Philippines, Portugal, Russian Federation, Serbia, Spain, Turkey, Ukraine	NR	Received effective antibacterial drug therapy for the index infection of HABP/VABP for >24 hours continuously, during the previous 72 hours	NR	Expected to survive for <72 hours	NR
Schmitt 2006 phase 3b [33] N = 221	Jan 1999–Dec 2001	Double-blind	Germany, Czech Republic, Hungary	NR	Received systemic antibacterial medication 24 hours before study start, unless a respiratory culture showed that a pathogen was resistant to that agent	Concurrent hemodialysis, peritoneal dialysis	NR	<8 or >25
Torres 2000 phase NR [34] N = 149	NR	Open-label	Spain	NR	NR	NR	NR	NR
West 2003 phase NR [36] N = 438	Dec 1997–Jun 2001	Open-label	United States, Canada	Center and patients’ need for intubation	NR	NR	NR	>35
Zanetti 2003 phase NR [39] N = 209	Apr 1997– May 1999	Open-label	Europe	NR	Treated with one of the study antibiotics within 4 weeks before randomization or with any investigational drug within 30 days before randomization	NR	High likelihood of death within 48 hours	NR

APACHE II, Acute Physiologic Assessment and Chronic Health Evaluation II; HABP, hospital-acquired bacterial pneumonia; NP, nosocomial pneumonia; NR, not reported; SLR, systematic literature review; VABP, ventilator-associated bacterial pneumonia.

Patient characteristics are reported in S3 Table. Patient baseline characteristics of HABP/VABP studies reporting. The mean age across studies ranged from 43.6 [22] to 68.4 years [33], with a predominant male population ranging from 50% [26] to 81% [25]. Baseline comorbidity data was not consistently reported across trials; however, it was broadly similar between trials when reported. Acute Physiologic Assessment and Chronic Health Evaluation II (APACHE II) scores were reported in 68% of treatment arms and ranged from mean (standard deviation) of 14.5 (4.0) to 20 (6.2). The ranges of days (mean [standard deviation]) reported for intensive care unit length of stay and for mechanical ventilation were 6.3 (5.1) to 25.5 (17.5) (n = 3 studies) and 4 (2.8) to 17 (2.4) (n = 4 studies), respectively. Pathogen distribution varied widely among the studies and are summarized in S4 Table. Distribution of baseline causative pathogen.

### Relevance

The SLR identified RCTs for the majority of standard-of-care agents approved for the treatment of HABP/VABP. When the totality of the HABP/VABP and ventilated HABP evidence was considered, regardless of outcome and/or analysis population, a connected network was possible based on interventions (S1 Table. SLR PICOTS criteria for study eligibility and S1 Fig. Full network). However, there were multiple differences in outcome definitions, as well as assessment time points and analyses populations, across the studies included (S5 Table. Clinical response analysis population and definitions and S6 Table. All-cause mortality analysis population and definitions). For example, the response of cure was variable and included but was not limited to the following definitions: remission of the clinical manifestations of pneumonia, remission of pneumonia-related signs and symptoms, or multiple conditions that must be met such as the patient completed treatment, did not require further antibacterial treatment, improved or did not progress according to chest radiograph, and showed recovery from acute infection.

Furthermore, the trial characteristics summarized in Table 1 also demonstrate diverse inclusion/exclusion criteria applied in each of the included studies. For example, some studies excluded patients with renal failure [22–25, 31], some allowed prior/current antibiotic use outside of the interventions [23–28, 30, 32, 33, 36, 37, 39], and there was variability in the requirement of intensive care unit admission [22–25, 27, 28, 39], and/or the requirement of mechanical ventilation [22–25, 27, 31, 34] or the requirement for minimal disease severity as indicated by an APACHE II score [25, 32, 33, 36, 37]. Heterogeneity in study criteria requirements and outcomes across the included RCTs potentially compromises the relevance of the study population and suitability of the connected network.

Lastly, newer agents intended for treatment of HABP/VABP caused by gram-negative bacteria are being developed for patients with highly resistant gram-negative infections and HTA decision-makers are considering their use in this clinical setting and circumstance. In this SLR, all studies included data from noninferiority RCTs in which respiratory isolates demonstrated susceptibility to at least one of the treatments. This has important implications for context, as any newer agents approved for HABP/VABP caused by gram-negative pathogens will ultimately be considered for patients with infections that are resistant to first-line treatment agents.

### Credibility

**Fig 2:**
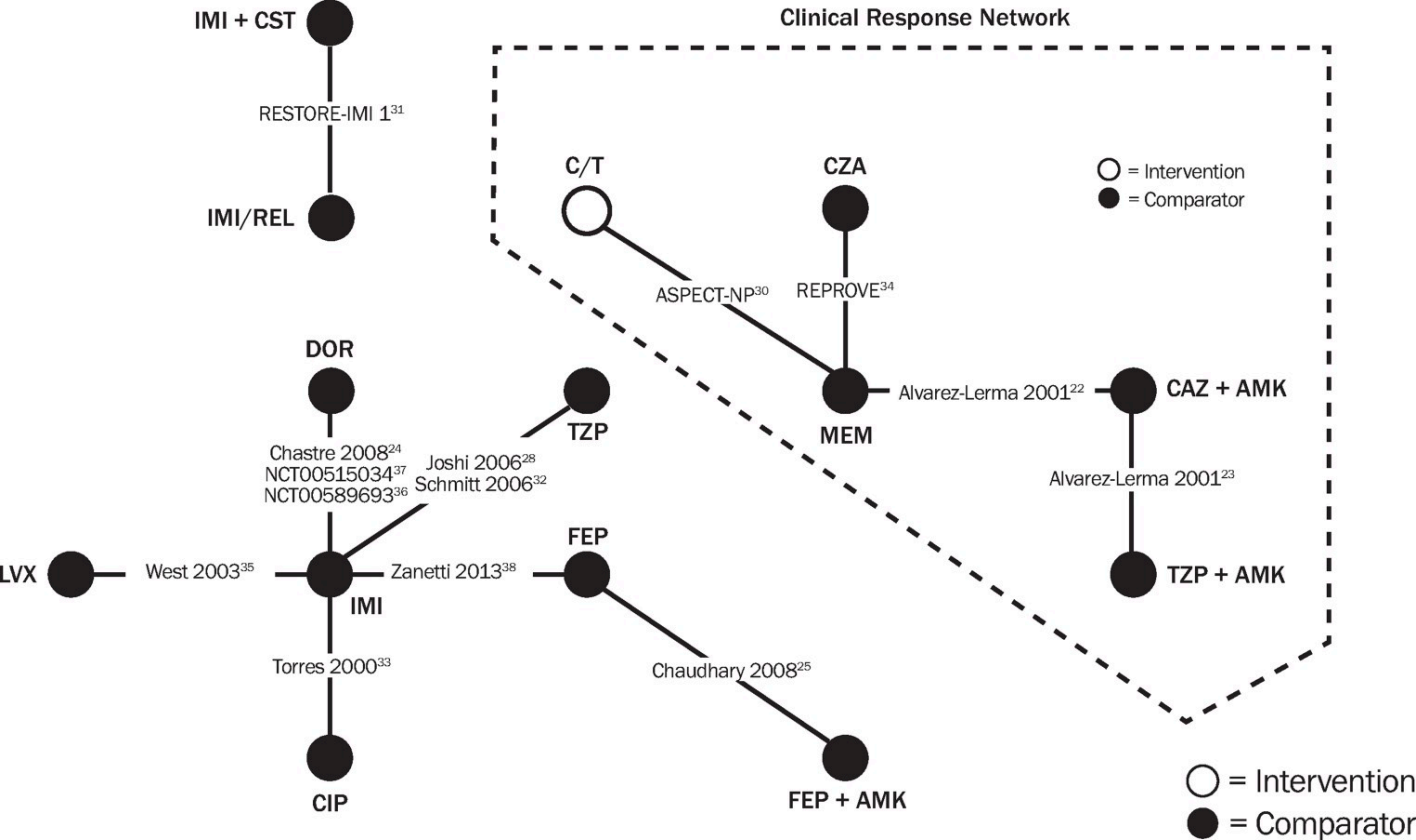
Networks resulting from credibility analyses of clinical response outcomes. The clinical response network on the right has the highest credibility.

The clinical response outcome was used to assess credibility in the network. Once applied, the large network collapsed except for four studies and five interventions, including ceftolozane/tazobactam connected (Fig 2, right side). This clinical response–connected network did not include closed loop(s) that were required to assess consistency (direct and indirect evidence). Comparisons of studies in the clinical response network showed that >70% of patients were ventilated at baseline in three of the four studies (Torres et al. reported 34%) and had generally high APACHE II scores (range of means, 14.7–17.5). The most common causative pathogens reported in the clinical response–connected network RCTs were *Pseudomonas aeruginosa* (range, 18.4–64.1% of patients) and *Klebsiella* spp. (range, 1.6–49%) (S4 Table. Distribution of baseline causative pathogen and S2 Fig. Proportion of patients with select pathogens in HABP/VABP in the clinical response-connected network).

Although all studies admitted patients with HABP/VABP, there were several differences across baseline patient characteristics, pathogen distribution, and treatment characteristics (S3 Table. Patient baseline characteristics of HABP/VABP studies reporting). Many of the baseline patient/pathogen characteristics that varied across RCTs have been recognized within the literature as effect modifiers [52] and these systematic differences have the potential to affect treatment outcomes. For example, patients who are ventilated and/or have a higher APACHE II score might be expected to have worse clinical outcomes relative to nonventilated patients with lower disease severity. Patients with *P*. *aeruginosa* tend to have lower response rates relative to Enterobacterales.

**Fig 3:**
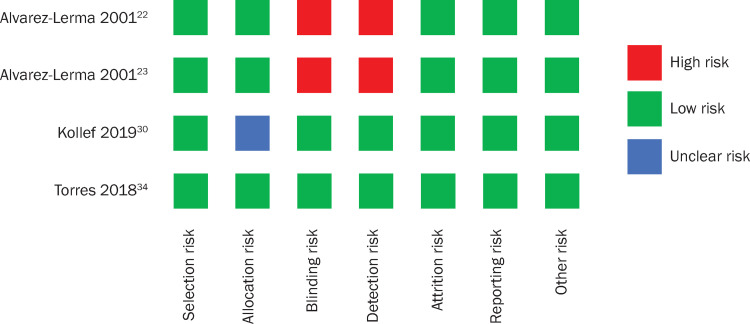
Risk of bias quality assessment using the Cochrane Collaboration risk-of-bias tool applied to the network of randomized-controlled trials reporting outcomes for clinical response.

Study quality as assessed with the Cochrane Collaboration risk-of-bias assessment tool demonstrated that among studies in the clinical response–connected network, the overall risk of bias across the 7 domains was considered low (Fig 3). High risk of bias was reported for two studies [23, 24]. One study reported unclear risk of bias for allocation, which was not clearly described in the published article [31].

Risk of bias assessment for all studies identified in the SLR are summarized in S7 Table. Cochrane Collaboration risk-of-bias assessment part 1 of 2, S8 Table. Cochrane Collaboration risk-of-bias assessment part 2 of 2, and S3 Fig. Cochrane Collaboration risk-of-bias tool summary graphic.

## Discussion

With increased attention on ITCs for informing health care policy in infectious diseases, we sought to determine if the available data on interventions from recent HABP/VABP RCTs were adequately feasible and reliable to conduct an NMA. Using data from an SLR, we examined the relevance and credibility of a potential NMA within the antibacterial therapeutic space for the indication of HABP/VABP. This review provides a point-by-point illustration of important considerations related to the potential use of NMAs for valid comparison and decision-making when evaluating agents for the treatment of adult patients with HABP/VABP caused by gram-negative pathogens. Overall, we believe that the available data from HABP/VABP RCTs do not provide support for a scientifically and clinically meaningful NMA from the perspective of the HTA decision-maker. Furthermore, we believe the issues that we identified in our review of the HABP/VABP NMA precludes its use in informing HTAs, payor decisions, guidelines development, and treatment protocol decisions for patients with HABP/VABP. This finding was consistent with the recent critical analysis of existing studies included in European Society of Clinical Microbiology and Infectious Diseases guidelines and endorsed by the European Society of Intensive Care Medicine, which also found that the heterogeneity of study designs, treatment schemes, and patient populations precluded a quantitative synthesis of data and the expert panel decided not to perform an NMA [53]. Furthermore, the shortcomings of the published data that evaluate antibacterial agents for approval in patients with HABP/VABP, and currently required clinical study designs, preclude worthwhile analysis and cannot accurately inform HTAs, payor decisions, guidelines development, and treatment protocol decisions.

With regards to credibility overall, the SLR identified highly relevant RCTs for most standard-of-care agents approved for the treatment of HABP/VABP. As the perspective of the review was based on an HTA decision-maker, we focused our credibility analysis on the clinical response outcome, and this resulted in a small but connected network. Due to the registrational intent of the identified studies, there was limited risk of publication bias or selective reporting. However, as a consequence of limited trials, there was a notable absence of closed loops within the clinical response–connected network and precluded the ability to assess consistency by means of direct and indirect evidence.

Although all RCTs included hospitalized adult patients with HABP/VABP, there were observed differences in outcomes, analysis populations, and pathogen distributions and these challenge the appropriateness of both relevance and credibility. Heterogenous study criteria were applied across the RCTs, and this resulted in distinctive differences in study populations and pathogen distributions at baseline. Similarly, a previous study that assessed the feasibility of an NMA based on published RCTs of antibacterial agents used to treat gram-negative complicated urinary tract infections concluded that heterogeneity of the evidence base renders NMA an impractical and unreliable method to make inferences of superiority of antibacterial agents [54]. In addition, some of the observed differences in this study have been recognized within the literature as effect modifiers [52] and would be expected to influence treatment effect. For example, patients who are critically ill, as determined by the need for ventilation and/or a high APACHE II score, might be expected to have worse clinical outcomes compared with patients who are not ventilated and/or have a lower APACHE II score. Further, severely ill patients were excluded from some studies, whereas other studies included these patients [25, 33, 36].

There were also several other notable findings in this analysis that were immediately apparent in the connected network assessment of the study characteristics and populations. Due to the limited number of studies identified, and that each intervention was supported by a single noninferiority study, it was not possible to explore the variability observed in key effect modifiers by means of regression analysis or the discard studies considered nonrelevant, as this would result in further breaks within the connected network. More importantly, noninferiority studies are not designed to show advantages of a treatment, but rather that it is no worse than a prespecified margin; the variability of this margin adds to the heterogeneity of the data.

Lastly, all studies included patients from noninferiority RCTs whose recovered pathogen had in vitro susceptibility to at least one of the treatments (ie, pathogen susceptible to drug). This has important implications for relevance (ie, context), as the newer agents approved for HABP/VABP caused by gram-negative pathogens are specifically developed for patients with infections resistant to first-line treatment agents. Recent susceptibility studies demonstrate varying susceptibility to a wide range of antibacterial agents [55–57]. Therefore, the applicability and use of noninferiority RCTs of susceptible pathogens to empirically support decision-making frameworks may be detrimental when trying to determine the value of novel antibacterial agents. Given the limitations associated with NMA of HABP/VABP RCTs, other types of studies, including regional microbiologic surveillance data and real-world comparative effectiveness studies that evaluate agents for patients with resistant infections should be considered when making formulary or reimbursement decisions on a national level, especially for patients with serious infections that are resistant to first-line therapies.

In addition to the significant limitations of HABP/VABP RCTs for NMA, some aspects of the methodology for selection of RCTs for this study may be considered a limitation of this analysis. Specifically, the relevance and credibility of the selections remains subjective despite use of Cochrane dual-reviewer methodology, PRISMA guidelines, and PICOTS criteria, which were employed to minimize bias in the selection of studies for this SLR.

## Conclusions

In summary, our appraisal of the available evidence using the ISPOR Task Force questionnaire for the clinical response outcome among adult patients with HABP/VABP does not support the conduct of a scientifically robust and clinically meaningful NMA. Although this data is vital to registration, there are significant limitations in these trials for HTAs, payor decisions, guidelines, and treatment protocol decisions. As new antibacterial agents emerge, the existing literature base available for comparisons will continue to grow; however, the same challenges that we identified here will remain and the feasibility of NMAs will need to be reassessed, notwithstanding external changes such as ecology, resistance, and changes in clinical practice. Further utilization and consistent inclusion of microbiologic and real-world evidence may provide a more thorough assessment of the value and place for novel antibacterial agents.

## Supporting information

S1 Checklist PRISMA 2020 for abstracts checklist. (DOCX)

S2 Checklist PRISMA 2020 checklist. (DOCX)

S1 File Study methodology. (PDF)

S1 Table SLR PICOTS criteria for study eligibility. (PDF)

S2 Table Detailed search strategy. (PDF)

S3 Table Patient baseline characteristics of HABP/VABP studies reporting. (PDF)

S4 Table Distribution of baseline causative pathogen. (PDF)

S5 Table Clinical response analysis population and definition. (PDF)

S6 Table All-cause mortality analysis populations and definitions. (PDF)

S7 Table Cochrane Collaboration risk-of-bias assessment part 1 of 2. (PDF)

S8 Table Cochrane Collaboration risk-of-bias assessment part 2 of 2. (PDF)

S1 Fig Full network. (PDF)

S2 Fig Proportion of patients with select pathogens in HABP/VABP in the clinical response–connected network. (PDF)

S3 Fig Cochrane Collaboration risk-of-bias tool summary graphic. (PDF)
