# Supplementary material for: Methods to appraise available evidence and adequacy of data from a systematic literature review to conduct a robust network meta-analysis of treatment options for patients with hospital-acquired or ventilator-associated bacterial pneumonia
Source: PLoS One. 2023 Jan 4;18(1):e0279844. doi: 10.1371/journal.pone.0279844 (PMC9812328; doi:10.1371/journal.pone.0279844)
Supplement: S1 File — (PDF) [file pone.0279844.s003.pdf]

Methods to appraise available evidence and adequacy of data from a systematic literature review to conduct a robust network meta-analysis of treatment options for patients with hospital-acquired or ventilator-associated bacterial pneumonia

Laura Puzniak[1#], Ryan Dillon[1]*, Thomas Lodise[2]

1 Merck & Co., Inc., Rahway, New Jersey, United States of America, **2** Department of Pharmacy Practice, Albany College of Pharmacy and Health Sciences, Albany, New York, United States of America

[#]LP was an employee of Merck & Co., Inc. at the time the study was conducted

*Corresponding author

E-mail: ryan.dillon@merck.com (RD)

Short title: Network meta-analysis HABP/VABP evidence appraisal

S1 File. Study methodology.

Systematic literature review (SLR) methodology

An SLR was conducted to obtain comprehensive and up-to-date data on September 27, 2018. This SLR was conducted using a standardized, thorough, and transparent method after Cochrane dual-reviewer methodology. The SLR protocol followed the Preferred Reporting Items for Systematic Reviews and Meta-Analyses protocol guidelines. The PICOTS (population, interventions, comparators, outcomes, timing, and setting) were included in the SLR (S1 Table).

The databases that were searched included MEDLINE®, Embase®, and the Cochrane Central Register of Controlled Trials via the Ovid® platform, with publications between 2000 and 2019. The search terms were related to the brand and generic names of antibacterial agents of interest and the hospital-acquired or ventilator-associated bacterial pneumonia disease area, and included terms and study design filters recommended by the Scottish Intercollegiate Guidelines Network [57] for identifying clinical trials in MEDLINE and Embase (S2 Table).

Study screening and quality assessment

Two independent reviewers conducted screening, data extraction, and study quality assessment using the Cochrane Collaboration risk-of-bias tool [18]; discordance between the two reviewers was resolved by a third who provided consensus.

Conference publications and other searches

To identify potentially eligible randomized-controlled trials not published at the time of search, ClinicalTrials.gov and conference proceedings (2018–2019) were searched manually.

Critical appraisal and quality assurance

Quality assessment of publications is displayed in S3 Fig.

Data extraction

Full-text studies found to be eligible were included for data extraction, including but not limited to study characteristics (ie, design, inclusion/exclusion criteria, sample size at baseline, and follow-up by intervention), participant characteristics (ie, demographic data, prior antibacterial treatment, comorbidities, causative pathogens), interventions characteristics (ie, active therapy, concomitant therapies, treatment dose and schedule, method of administration), and outcomes (ie, analysis population, clinical outcomes, adverse events).
