# Supplementary material for: Methods to appraise available evidence and adequacy of data from a systematic literature review to conduct a robust network meta-analysis of treatment options for patients with hospital-acquired or ventilator-associated bacterial pneumonia
Source: PLoS One. 2023 Jan 4;18(1):e0279844. doi: 10.1371/journal.pone.0279844 (PMC9812328; doi:10.1371/journal.pone.0279844)
Supplement: S1 Table — (PDF) [file pone.0279844.s004.pdf]

Methods to appraise available evidence and adequacy of data from a systematic literature review to conduct a robust network meta-analysis of treatment options for patients with hospital-acquired or ventilator-associated bacterial pneumonia

Laura Puzniak[1#], Ryan Dillon[1]*, Thomas Lodise[2]

1 Merck & Co., Inc., Rahway, New Jersey, United States of America, **2** Department of Pharmacy Practice, Albany College of Pharmacy and Health Sciences, Albany, New York, United States of America

[#]LP was an employee of Merck & Co., Inc. at the time the study was conducted

*Corresponding author

E-mail: ryan.dillon@merck.com (RD)

Short title: Network meta-analysis HABP/VABP evidence appraisal

S1 Table. SLR PICOTS criteria for study eligibility.

Criteria	Inclusion criteria	Exclusion criteria
**Population**	• Adult or pediatric patients experiencing gram-negative HABP or VABP	• Patients experiencing gram-positive HABP or VABP<br>• Patients not experiencing any HABP or VABP
**Interventions**	• Ceftolozane/tazobactam<br>• Amikacin<br>• Cefepime<br>• Cefiderocol<br>• Ceftazidime<br>• Ceftazidime/avibactam<br>• Ceftriaxone<br>• Ciprofloxacin<br>• Colistin<br>• Doripenem<br>• Ertapenem<br>• Gentamicin<br>• Imipenem<br>• Imipenem/cilastatin<br>• Imipenem/relebactam<br>• Levofloxacin<br>• Meropenem<br>• Meropenem and vaborbactam (mero-vabor)<br>• Piperacillin/tazobactam<br>• Plazomicin<br>• Polymyxin<br>• Prulifloxacin<br>• Tobramycin	• Not an intervention of interest<br>• Patients using inhaled treatment interventions
**Comparisons**	• Any intervention of interest listed above under "Interventions"<br>• Placebo<br>• Any other antibiotic	• Not a comparator of interest<br>• Patients using inhaled treatment interventions
**Outcomes**	• Clinical cure or success, and definition, if reported<br>• Microbiologic cure or eradication<br>• Composite (clinical and microbiologic) cure<br>• Mortality<br>• Mechanical ventilation days<br>• Hospital length of stay<br>• ICU length of stay	• Not reporting an outcome of interest

	• Readmission<br>• Relapse	
**Time**	• Published between 2000 and present	• Published before 2000
**Study design**	• Randomized-controlled trials	• Not reporting on a randomized-controlled trial
**Other**	• English language publications	• Non-English language publications

HABP, hospital-acquired bacterial pneumonia; ICU, intensive care unit; PICOTS, population, interventions, comparators, outcomes, timing, and setting; SLR, systematic literature review; VABP, ventilator-associated bacterial pneumonia.
