# Supplementary material for: Methods to appraise available evidence and adequacy of data from a systematic literature review to conduct a robust network meta-analysis of treatment options for patients with hospital-acquired or ventilator-associated bacterial pneumonia
Source: PLoS One. 2023 Jan 4;18(1):e0279844. doi: 10.1371/journal.pone.0279844 (PMC9812328; doi:10.1371/journal.pone.0279844)
Supplement: S2 Table — (PDF) [file pone.0279844.s005.pdf]

Methods to appraise available evidence and adequacy of data from a systematic literature review to conduct a robust network meta-analysis of treatment options for patients with hospital-acquired or ventilator-associated bacterial pneumonia

Laura Puzniak[1#], Ryan Dillon[1]*, Thomas Lodise[2]

1 Merck & Co., Inc., Rahway, New Jersey, United States of America, **2** Department of Pharmacy Practice, Albany College of Pharmacy and Health Sciences, Albany, New York, United States of America

[#]LP was an employee of Merck & Co., Inc. at the time the study was conducted

*Corresponding author

E-mail: ryan.dillon@merck.com (RD)

Short title: Network meta-analysis HABP/VABP evidence appraisal

S2 Table. Detailed search strategy.

**Embase in Ovid, 1974–2018, executed September 27, 2018**		
**Search number**	**Search term**	**Number of hits**
1	Clinical Trial/	949,889
2	Randomized Controlled Trial/	513,634
3	controlled clinical trial/	457,965
4	multicenter study/	194,205
5	Phase 3 clinical trial/	35,674
6	Phase 4 clinical trial/	3,083
7	exp RANDOMIZATION/	79,618
8	Single Blind Procedure/	32,399
9	Double Blind Procedure/	152,931
10	Crossover Procedure/	56,521
11	PLACEBO/	323,113
12	randomi?ed controlled trial$.tw.	185,951
13	rct.tw.	29,424
14	(random$ adj2 allocat$).tw.	37,528
15	single blind$.tw.	21,569
16	double blind$.tw.	190,283
17	((treble or triple) adj blind$).tw.	835
18	placebo$.tw.	277,381
19	Prospective Study/	469,836
20	or/1-19	2,017,320
21	Case Study/	56,327
22	case report.tw.	362,426
23	abstract report/ or letter/	1,070,232
24	Conference proceeding.pt.	0
25	Conference abstract.pt.	3,156,716
26	Editorial.pt.	576,421
27	Letter.pt.	1,031,422
28	Note.pt.	723,321
29	or/21-28	5,894,245
30	20 not 29	1,542,748
31	exp pneumonia, bacterial/	27,430
32	(bacterial AND pneumonia).mp.	44,889
33	31 or 32	56,297
34	((hospital OR ventilator OR nosocomial) AND pneumonia).mp.	70,710
35	33 AND 34	14,517

36	exp pneumonia, ventilator-associated/	9,046
37	35 or 36	21,466
38	(meropenem or Merrem or Penem or Ronem or SM 7338 or SM-7338).mp.	28,666
39	(ceftolozane or 'CXA-201' or 'cb-500,201' OR cb500201 OR 'cxa-201' or cxa201 or 'cxa 101 plus tazobactam' or 'ceftolozane tazobactam' or 'ceftolozane plus tazobactam' or 'ceftolozane-tazobactam' or (ceftolozane adj2 tazobactam)).mp.	520
40	piperacillin plus tazobactam/	22,478
41	('piperacillin plus tazobactam' or (Piperacillin AND tazobactam) or (piperacillin adj2 tazobactam) or 'piperacillin-tazobactam' or Tazocin or Zosyn or tazopril or tazobac or tazonam or tazocillin or tazocilline or tazomax or tazocel).mp.	25,888
42	(cefepim or cefepime or cefepime hyrdrochloride or Quadrocef or Maxipime or Axepim or BMY 28142 or BMY-28142).ti,ab,kw.	4,970
43	exp levofloxacin/	31,444
44	(levofloxacin or Levaquin or Tavanic or iquix or mosardal or nofaxin or levox or 'cravit aphtalmic' or levokacin or lesacin or tavanic or quixin or cravit or reskuin or levaquin or floxel or leroxacin or volequin or elequine).mp.	32,476
45	exp ceftazidime/	37,466
46	(ceftazidime OR Fortum or LY-139381 OR LY 139381 OR LY139381 OR tazidime OR Fortaz OR GR-20263 OR GR 20263 OR GR20263).mp.	38,869
47	exp imipenem/	34,173
48	(imipenem OR Primaxin or MK-0787 OR MK 0787 OR MK0787 or N-Formimidoylthienamycin OR N Formimidoylthienamycin).mp.	38,933
49	ciprofloxacin/	89,167
50	(ciprofloxacin or Ciloxan or Cipro or Neofloxin).mp.	92,385
51	exp prulifloxacin/	472
52	(prulifloxacin or Quisnon or Unidrox or Prixina or Glimbax).mp.	483
53	exp ertapenem/	6,093
54	(ertapenem or Invanz).mp.	6,226
55	exp doripenem/	2,341
56	(doripenem or Doribax or Finibax).mp.	2,388
57	exp ceftriaxone/	52,857
58	(ceftriaxone or Rocephin or Epicephin).mp.	54,334
59	exp amikacin/	42,084

60	(amikacin OR Yectamid OR Amikacina OR Amikafur OR amikalem or Amikason's OR Amikin OR Biklin OR Biclin OR Amiklin OR Amukin OR BB-K8 or BB K8 OR BBK8 OR BB-K 8 OR BB K 8 OR BBK 8 OR gamikal OR kanbine OR oprad).mp	43,495
61	exp gentamicins/	97,970
62	(gentamycin OR garamycin OR gentacyclo OR Gentavet OR genticin OR G-Myticin OR Gmyticin OR G Myticin OR Gentamicin OR Gentamycin).mp.	102,779
63	exp tobramycin/	30,439
64	(tobramycin OR obracin OR tobracin OR brulamycin OR nebcin OR nebicin OR nebramycin).mp.	32,208
65	exp plazomicin/	176
66	(plazomicin or Zemdri).mp.	183
67	('colistin plus polymyxin' or 'colistin-polymyxin' or (colistin adj2 polymyxin)).mp.	370
68	exp colistin/	14,998
69	("Colisticin" or "Polymyxin E" or "Colimycin" or "Coly-Mycin" or "Totazina" or "Colistin Sulfate" or "Sulfate, Colistin").mp.	749
70	exp polymyxins/	5,715
71	(“Polymyxin” or “Polymyxin M”).mp.	16,738
72	exp cefiderocol/	47
73	or/38-72	286,844
74	30 and 37 and 73	1,141
75	limit 74 to yr="2000-Current"	1,045
**MEDLINE in Ovid, 1946–2018, executed September 27, 2018**		
**Search number**	**Search term**	**Number of hits**
1	Randomized Controlled Trials as Topic/	118,569
2	randomized controlled trial/	468,737
3	Random Allocation/	95,976
4	Double Blind Method/	147,619
5	Single Blind Method/	25,713
6	clinical trial/	512,486
7	clinical trial, phase i.pt	18,390
8	clinical trial, phase ii.pt	29,645
9	clinical trial, phase iii.pt	14,183
10	clinical trial, phase iv.pt	1,594
11	controlled clinical trial.pt	92,661
12	randomized controlled trial.pt	468,737
13	multicenter study.pt	239,474
14	clinical trial.pt	512,486

15	exp Clinical Trials as topic/	317,783
16	or/1-15	1,256,543
17	(clinical adj trial$).tw	315,644
18	((singl$ or doubl$ or treb$ or tripl$) adj (blind$3 or mask$3)).tw	159,000
19	PLACEBOS/	34,073
20	placebo$.tw	198,184
21	randomly allocated.tw	24,902
22	(allocated adj2 random$).tw	27,978
23	or/17-22	565,124
24	16 or 23	1,484,868
25	case report.tw	276,679
26	letter/	1,000,335
27	historical article/	347,230
28	or/25-27	1,609,841
29	24 not 28	1,451,138
30	exp pneumonia, bacterial/	20,520
31	(bacterial AND pneumonia).mp.	36,325
32	30 or 31	41,897
33	((hospital OR ventilator OR nosocomial) AND pneumonia).mp.	29,441
34	32 AND 33	9,860
35	exp pneumonia, ventilator-associated/	2,999
36	34 or 35	11,617
37	(meropenem or Merrem or Penem or Ronem or SM 7338 or SM-7338).mp.	6,220
38	(ceftolozane or 'CXA-201' or 'cb-500,201' OR cb500201 OR 'cxa-201' or cxa201 or 'cxa 101 plus tazobactam' or 'ceftolozane tazobactam' or 'ceftolozane plus tazobactam' or 'ceftolozane-tazobactam' or (ceftolozane adj2 tazobactam)).mp.	277
39	('piperacillin plus tazobactam' or (Piperacillin AND tazobactam) or (piperacillin adj2 tazobactam) or 'piperacillin-tazobactam' or Tazocin or Zosyn or tazopril or tazobac or tazonam or tazocillin or tazocilline or tazomax or tazocel).mp.	3,811
40	(cefepim or cefepime or cefepime hyrdrochloride or Quadrocef or Maxipime or Axepim or BMY 28142 or BMY-28142).ti,ab,kw.	3,020
41	exp levofloxacin/	2,982
42	(levofloxacin or Levaquin or Tavanic or iquix or mosardal or nofaxin or levox or 'cravit aphtalmic' or levokacin or lesacin or tavanic or quixin or cravit or reskuin or	6,935

	levaquin or floxel or leroxacin or volequin or elequine).mp.	
43	exp ceftazidime/	3,648
44	(ceftazidime OR Fortum or LY-139381 OR LY 139381 OR LY139381 OR tazidime OR Fortaz OR GR-20263 OR GR 20263 OR GR20263).mp.	8,896
45	exp imipenem/	3,867
46	(imipenem OR Primaxin or MK-0787 OR MK 0787 OR MK0787 or N-Formimidoylthienamycin OR N Formimidoylthienamycin).mp.	10,224
47	ciprofloxacin/	12,232
48	(ciprofloxacin or Ciloxan or Cipro or Neofloxin).mp.	25,826
49	(prulifloxacin or Quisnon or Unidrox or Prixina or Glimbax).mp.	142
50	(ertapenem or Invanz).mp.	1,369
51	(doripenem or Doribax or Finibax).mp.	600
52	exp ceftriaxone/	5,517
53	(ceftriaxone or Rocephin or Epicephin).mp.	11,330
54	exp amikacin/	3,924
55	(amikacin OR Yectamid OR Amikacina OR Amikafur OR amikalem or Amikason's OR Amikin OR Biklin OR Biclin OR Amiklin OR Amukin OR BB-K8 or BB K8 OR BBK8 OR BB-K 8 OR BB K 8 OR BBK 8 OR gamikal OR kanbine OR oprad).mp	9,488
56	exp gentamicins/	18,372
57	(gentamycin OR garamycin OR gentacyclo OR Gentavet OR genticin OR G-Myticin OR Gmyticin OR G Myticin OR Gentamicin OR Gentamycin).mp.	25,467
58	exp tobramycin/	4,095
59	(tobramycin OR obracin OR tobracin OR brulamycin OR nebcin OR nebicin OR nebramycin).mp.	7,509
60	(plazomicin or Zemdri).mp.	57
61	('colistin plus polymyxin' or 'colistin-polymyxin' or (colistin adj2 polymyxin)).mp.	310
62	exp colistin/	3,582
63	("Colisticin" or "Polymyxin E" or "Colimycin" or "Coly-Mycin" or "Totazina" or "Colistin Sulfate" or "Sulfate, Colistin").mp.	711
64	exp polymyxins/	8,571
65	(“Polymyxin” or “Polymyxin M”).mp.	7,330
66	cefiderocol.mp.	28
67	or/37-66	99,993
68	29 and 36 and 67	378
69	limit 68 to yr="2000-Current"	306

Cochrane Central Register of Controlled Trials in Ovid, executed September 27, 2018		
**Search number**	**Search term**	**Number of hits**
1	exp pneumonia, bacterial/	710
2	(bacterial AND pneumonia).mp.	2,153
3	1 or 2	2,249
4	((hospital OR ventilator OR nosocomial) AND pneumonia).mp.	3,561
5	3 AND 4	853
6	exp pneumonia, ventilator-associated/	319
7	5 or 6	1,041
8	(meropenem or Merrem or Penem or Ronem or SM 7338 or SM-7338).mp.	518
9	(ceftolozane or 'CXA-201' or 'cb-500,201' OR cb500201 OR 'cxa-201' or cxa201 or 'cxa 101 plus tazobactam' or 'ceftolozane tazobactam' or 'ceftolozane plus tazobactam' or 'ceftolozane-tazobactam' or (ceftolozane adj2 tazobactam)).mp.	31
10	('piperacillin plus tazobactam' or (Piperacillin AND tazobactam) or (piperacillin adj2 tazobactam) or 'piperacillin-tazobactam' or Tazocin or Zosyn or tazopril or tazobac or tazonam or tazocillin or tazocilline or tazomax or tazocel).mp.	498
11	(cefepim or cefepime or cefepime hyrdrochloride or Quadrocef or Maxipime or Axepim or BMY 28142 or BMY-28142).ti,ab,kw.	262
12	exp levofloxacin/	524
13	(levofloxacin or Levaquin or Tavanic or iquix or mosardal or nofaxin or levox or 'cravit aphtalmic' or levokacin or lesacin or tavanic or quixin or cravit or reskuin or levaquin or floxel or leroxacin or volequin or elequine).mp.	1,284
14	exp ceftazidime/	446
15	(ceftazidime OR Fortum or LY-139381 OR LY 139381 OR LY139381 OR tazidime OR Fortaz OR GR-20263 OR GR 20263 OR GR20263).mp.	994
16	exp imipenem/	282
17	(imipenem OR Primaxin or MK-0787 OR MK 0787 OR MK0787 or N-Formimidoylthienamycin OR N Formimidoylthienamycin).mp.	630
18	ciprofloxacin/	1,057
19	(ciprofloxacin or Ciloxan or Cipro or Neofloxin).mp.	2,376
20	(prulifloxacin or Quisnon or Unidrox or Prixina or Glimbax).mp.	46

21	(ertapenem or Invanz).mp.	161
22	(doripenem or Doribax or Finibax).mp.	69
23	exp ceftriaxone/	662
24	(ceftriaxone or Rocephin or Epicephin).mp.	1,426
25	exp amikacin/	349
26	(amikacin OR Yectamid OR Amikacina OR Amikafur OR amikalem or Amikason's OR Amikin OR Biklin OR Biclin OR Amiklin OR Amukin OR BB-K8 or BB K8 OR BBK8 OR BB-K 8 OR BB K 8 OR BBK 8 OR gamikal OR kanbine OR oprad).mp	868
27	exp gentamicins/	1,143
28	(gentamycin OR garamycin OR gentacyclo OR Gentavet OR genticin OR G-Myticin OR Gmyticin OR G Myticin OR Gentamicin OR Gentamycin).mp.	1,904
29	exp tobramycin/	567
30	(tobramycin OR obracin OR tobracin OR brulamycin OR nebcin OR nebicin OR nebramycin).mp.	1,331
31	(plazomicin or Zemdri).mp.	6
32	('colistin plus polymyxin' or 'colistin-polymyxin' or (colistin adj2 polymyxin)).mp.	5
33	exp colistin/	148
34	("Colisticin" or "Polymyxin E" or "Colimycin" or "Coly-Mycin" or "Totazina" or "Colistin Sulfate" or "Sulfate, Colistin").mp.	60
35	exp polymyxins/	366
36	(“Polymyxin” or “Polymyxin M”).mp.	395
37	cefiderocol.mp.	4
38	or/8-37	94,639,849
39	7 and 38	288
40	limit 39 to yr="2000-Current"	231
