# Supplementary material for: Methods to appraise available evidence and adequacy of data from a systematic literature review to conduct a robust network meta-analysis of treatment options for patients with hospital-acquired or ventilator-associated bacterial pneumonia
Source: PLoS One. 2023 Jan 4;18(1):e0279844. doi: 10.1371/journal.pone.0279844 (PMC9812328; doi:10.1371/journal.pone.0279844)
Supplement: S3 Table — (PDF) [file pone.0279844.s006.pdf]

Methods to appraise available evidence and adequacy of data from a systematic literature review to conduct a robust network meta-analysis of treatment options for patients with hospital-acquired or ventilator-associated bacterial pneumonia

Laura Puzniak[1#], Ryan Dillon[1]*, Thomas Lodise[2]

1 Merck & Co., Inc., Rahway, New Jersey, United States of America, **2** Department of Pharmacy Practice, Albany College of Pharmacy and Health Sciences, Albany, New York, United States of America

[#]LP was an employee of Merck & Co., Inc. at the time the study was conducted

*Corresponding author

E-mail: ryan.dillon@merck.com (RD)

Short title: Network meta-analysis HABP/VABP evidence appraisal

S3 Table1. Patient baseline characteristics of HABP/VABP studies reporting.

Study	Intervention	N	Mean (SD) age, y	Male, n (%)	Prior AB, n (%)	HABP/VABP, n (%)	VABP, n (%)	APACHE II score (SD)	SOFA score (SD)	ICU LOS (SD), days	Mechanical ventilation (SD), days
		**Studies reporting clinical response (n = 4) within ASPECT NP–connected network**									
Alvarez Lerma 2001 [22]	Meropenem	69	61.5 (13.7)	47 (68.1)	9 (13.0)	69 (100)	69 (100)	16.5 (5.7)	–	24.2 (14.9)	16.5 (11.4)
Alvarez Lerma 2001 [22]	Ceftazidime + amikacin	71	62.3 (15.7)	56 (78.9)	7 (9.9)	71 (100)	71 (100)	16.6 (6.0)	–	25.5 (17.5)	17 (12.4)
Alvarez-Lerma 2001 [23]	Piperacillin/ tazobactam + amikacin	88	57.1 (17)	64 (72.7)	43 (48.9)	88 (100)	75 (85.2)	16.5 (6.6)	–	–	–
Alvarez-Lerma 2001 [23]	Ceftazidime + Amikacin	36	60.5 (20.0)	26 (72.2)	21 (58.3)	36 (100)	31 (86.1)	16.9 (6.5)	–	–	–
ASPECT-NP [30]	Ceftolozane/ tazobactam	362	60.5 (16.7)	262 (72.4)	53 (14.6)	362 (100)	263 (72.7)	17.5 (5.2)	–	–	–
ASPECT-NP [30]	Meropenem	364	59.5 (17.2)	255 (70.1)	40 (11)	364 (100)	256 (70.3)	17.4 (5.7)	–	–	–
REPROVE [34]	Ceftazidime/ avibactam	356	62.1 (16.6)	268 (75.3)	224 (62.9)	356 (100)	118 (33.1)	14.5 (4.0)	–	–	–
REPROVE [34]	Meropenem	370	61.9 (17.4)	274 (74.1)	253 (68.4)	370 (100)	128 (34.6)	14.9 (4.0)	–	–	–
		**Remaining studies reporting HABP/VABP meeting SLR eligibility criteria**									
Ahmed 2007 [21]	Cefepime + levofloxacin	46	45.2 (5.1)	25 (54.3)	–	46 (100)	46 (100)	18 (2.0)	–	–	5 (1.4)
Ahmed 2007 [21]	Piperacillin/tazobactam + amikacin	47	43.6 (6.2)	28 (59.6)	–	47 (100)	47 (100)	16 (3.0)	–	–	4 (2.8)
Chastre 2008 [24]	Doripenem	126	50.7 (19.6)	102 (81.0)	–	126 (100)	126 (100)	–	–	–	–
Chastre 2008 [24]	Imipenem/cilastatin	122	50.3 (19.0)	91 (74.6)	–	122 (100)	122 (100)	–	–	–	–
Chaudhary 2008 [25]	Cefepime + amikacin	100	–	62 (62.0)	–	100 (100)	–	–	–	–	–
Chaudhary 2008 [25]	Cefepime	100	–	50 (50.0)	–	100 (100)	–	–	–	–	–

Study	Intervention	N	Mean (SD) age, y	Male, n (%)	Prior AB, n (%)	HABP/VABP, n (%)	VABP, n (%)	APACHE II score (SD)	SOFA score (SD)	ICU LOS (SD), days	Mechanical ventilation (SD), days
Damas 2006 [26]	Cefepime	20	53.1 (22.1)	13 (65.0)	–	20 (100)	20 (100)	17.1 (4.6)	6.9	–	7.2 (6.1)
Damas 2006 [26]	Cefepime + amikacin	19	64.7 (19.1)	10 (52.6)	–	19 (100)	19 (100)	14.6 (6.8)	7	–	6.5 (2.1)
Damas 2006 [26]	Cefepime + levofloxacin	20	59.2 (14.8)	15 (75.0)	–	20 (100)	20 (100)	16.5 (6.4)	7.3	–	9.4 (10.7)
Heyland 2008 [27]	Meropenem + ciprofloxacin	369	59.1 (17.9)	261 (70.7)	–	369 (100)	369 (100)	19.9 (6.4)	–	8 (4.9)	7.8 (4.9)
Heyland 2008 [27]	Meropenem	370	58.9 (17.7)	251 (67.8)	–	370 (100)	370 (100)	20 (6.2)	–	7.8 (5.5)	7.7 (5.7)
Joshi 2006 [28]	Piperacillin/ tazobactam	222	53.2 (19.1)	173 (78)	–	222 (100)	–	13.9	–	–	–
Joshi 2006 [28]	Imipenem/ cilastatin	215	52.7 (20.9)	138 (64)	–	215 (100)	–	13.0	–	–	–
NCT00515034 [37]	Doripenem	49	–	37 (75.5)	–	49 (100)	49 (100)	–	–	–	–
NCT00515034 [37]	Imipenem/cilastatin	15	–	9 (60.0)	–	15 (100)	15 (100)	–	–	–	–
NCT00589693 [36]	Doripenem	115	57.5 (16.5)	72 (62.6)	10 (14.5)	115 (100)	115 (100)	–	6 (2.7)	–	–
NCT00589693 [36]	Imipenem/cilastatin	112	54.6 (18.5)	75 (67.0)	11 (14.1)	112 (100)	112 (100)	–	5.5 (2.4)	–	–
RESTORE-IMI 1 [31]	Imipenem/cilastatin/ relebactam	21	59 (19–75)[a]	13 (61.9)	14 (66.7)	8 (38.1)	7 (33.3)	–	–	–	–
RESTORE-IMI 1 [31]	Imipenem/cilastatin + colistin	10	61 (49–80)[a]	7 (70.0)	7 (70.0)	3 (30.0)	2 (20.0)	–	–	–	–
RESTORE-IMI 2 [50]	Imipenem/cilastatin/ relebactam	264	60.5 (16.9)	178 (67.4)	–	–	–	–	–	–	–
RESTORE-IMI 2 [50]	Piperacillin/ tazobactam	267	58.8 (18.4)	189 (70.8)	–	–	–	–	–	–	–

Study	Intervention	N	Mean (SD) age, y	Male, n (%)	Prior AB, n (%)	HABP/VABP, n (%)	VABP, n (%)	APACHE II score (SD)	SOFA score (SD)	ICU LOS (SD), days	Mechanical ventilation (SD), days
Schmitt 2006 [32]	Piperacillin/tazobactam	110	68.4 (13.7)	77 (70.0)	–	110 (100)	–	13.5 (4.2)	–	–	–
Schmitt 2006 [32]	Imipenem/ cilastatin	111	65.7 (13.8)	64 (57.7)	–	111 (100)	–	13.3 (4.3)	–	–	–
Torres 2000 [33]	Ciprofloxacin	72	60 (16)	53 (73.6)	–	72 (100)	–	13.9 (7.7)	–	–	–
Torres 2000 [33]	Imipenem/ cilastatin	77	62 (16)	57 (74.0)	–	77 (100)	–	14.7 (7.6)	–	–	–
West 2003 [35]	Levofloxacin	220	55.8 (20.0)	161 (73.2)	–	220 (100)	157 (71.4)	15 (5.8)	–	–	–
West 2003 [35]	Imipenem/ cilastatin	218	55.5 (20.1)	154 (70.6)	–	218 (100)	154 (70.6)	14.8 (6.0)	–	–	–
Zanetti 2003 [38]	Cefepime	108	55 (18.0)	72 (66.7)	86 (79.6)	107 (100)	71 (66.4)	15.4 (6.6)	–	6.3 (5.1)	–
Zanetti 2003 [38]	Imipenem/ cilastatin	101	53 (18.0)	67 (66.3)	85 (84.2)	101 (100)	67 (66.3)	14.8 (6.7)	–	6.5 (5.8)	–

AB, antibacterial; APACHE II, Acute Physiologic Assessment and Chronic Health Evaluation II; HABP, hospital-acquired bacterial pneumonia; ICU, intensive care unit; LOS, length of stay; SD, standard deviation; SOFA, Sequential Organ Failure Assessment; SLR, systematic literature review; VABP, ventilator-associated bacterial pneumonia.

[a]Median (range).
