# Supplementary material for: Methods to appraise available evidence and adequacy of data from a systematic literature review to conduct a robust network meta-analysis of treatment options for patients with hospital-acquired or ventilator-associated bacterial pneumonia
Source: PLoS One. 2023 Jan 4;18(1):e0279844. doi: 10.1371/journal.pone.0279844 (PMC9812328; doi:10.1371/journal.pone.0279844)
Supplement: S4 Table — (PDF) [file pone.0279844.s007.pdf]

Methods to appraise available evidence and adequacy of data from a systematic literature review to conduct a robust network meta-analysis of treatment options for patients with hospital-acquired or ventilator-associated bacterial pneumonia

Laura Puzniak[1#], Ryan Dillon[1]*, Thomas Lodise[2]

1 Merck & Co., Inc., Rahway, New Jersey, United States of America, **2** Department of Pharmacy Practice, Albany College of Pharmacy and Health Sciences, Albany, New York, United States of America

[#]LP was an employee of Merck & Co., Inc. at the time the study was conducted

*Corresponding author

E-mail: ryan.dillon@merck.com (RD)

Short title: Network meta-analysis HABP/VABP evidence appraisal

S4 Table. Distribution of baseline causative pathogen.

Study	Intervention	Denominator	N	*Pseudomonas aeruginosa*, n (%)	*Acinetobacter baumannii*, n (%)	*Haemophilus influenzae*, n (%)	*Escherichia coli*, n (%)	*Klebsiella* spp., n (%)	*Enterobacter* spp., n (%)	*Proteus mirabilis*, n (%)	*Serratia marcescens*, n (%)
**Studies reporting clinical response (n = 4) within ASPECT NP–connected network**											
Alvarez Lerma 2001 [22]	Meropenem	By total bacteria	76	14 (18.4)	3 (3.9)	9 (11.8)	6 (7.9)	7 (9.2)	7 (9.2)	4 (5.3)	0 (0)
Alvarez Lerma 2001 [22]	Ceftazidime + amikacin	By total bacteria	65	13 (20.0)	2 (3.1)	17 (26.2)	3 (4.6)	2 (3.1)	4 (6.2)	2 (3.1)	2 (3.1)
Alvarez-Lerma 2001 [23]	Piperacillin/ tazobactam + amikacin	By total bacteria	64	41 (64.1)	2 (3.1)	11 (17.2)	5 (7.8)	1 (1.6)	2 (3.1)	3 (4.7)	3 (4.7)
Alvarez-Lerma 2001 [23]	Ceftazidime + amikacin	By total bacteria	29	16 (55.2)	2 (6.9)	3 (10.3)	2 (6.9)	1 (3.4)	2 (6.9)	0 (0)	1 (3.4)
ASPECT-NP [30]	Ceftolozane/tazobactam	By total gram-negative bacteria	113	29 (25.7)	6 (5.3)	12 (10.6)	23 (20.4)	50 (44.2)	7 (6.2)	11 (9.7)	5 (4.4)
ASPECT-NP [30]	Meropenem	By total gram-negative bacteria	117	38 (32.5)	5 (4.3)	8 (6.8)	23 (19.7)	55 (47.0)	8 (6.8)	10 (8.5)	6 (5.1)
REPROVE	Ceftazidime/avibactam	By patient	125	42 (33.6)	–	11 (8.8)	11 (8.8)	37	27 (21.6)	11 (8.8)	12 (9.6)
REPROVE	Meropenem	By patient	131	35 (26.7)	–	13 (9.9)	18 (13.7)	49	16 (12.2)	8 (6.1)	8 (6.1)
**Remaining studies reporting HABP/VABP meeting SLR eligibility criteria**											
Ahmed 2007 [21]	Cefepime + levofloxacin	By patient	47	20 (42.6)	3 (6.4)	–	7 (14.9)	2 (4.3)	–	–	–
Ahmed 2007 [21]	Piperacillin/ tazobactam + amikacin	By patient	46	17 (37.0)	2 (4.3)	–	10 (21.7)	2 (4.3)	–	–	–
Chastre 2008 [24]	Doripenem	By total gram-	224	30 (13.4)	15 (6.7)	47 (21.0)	18 (8.0)	34 (15.2)	37 (16.5)	6 (2.7)	12 (5.4)

Study	Intervention	Denominator	N	*Pseudomonas aeruginosa*, n (%)	*Acinetobacter baumannii*, n (%)	*Haemophilus influenzae*, n *(%)*	*Escherichia coli*, n (%)	*Klebsiella* spp., n (%)	*Enterobacter* spp., n (%)	*Proteus mirabilis*, n (%)	*Serratia marcescens*, n (%)
		negative bacteria									
Chastre 2008 [24]	Imipenem/cilastatin	By total gram-negative bacteria	236	26 (11.0)	16 (6.8)	55 (23.3)	30 (12.7)	31 (13.1)	22 (9.3)	8 (3.4)	8 (3.4)
Chaudhary 2008 [25]	Cefepime + amikacin	By patient	61	24 (39.3)	–	–	–	–	–	–	–
Chaudhary 2008 [25]	Cefepime	By patient	60	23 (38.3)	–	–	–	–	–	–	–
Damas 2006 [26]	Cefepime	By total bacteria	20	NA (23.3)	NA (3.3)	NA (6.7)	NA (10.0)	NA (10.0)	NA (10.0)	NA (3.3)	NA (6.7)
Damas 2006 [26]	Cefepime + amikacin	By total bacteria	19	NA (8.3)	NA (4.2)	NA (4.2)	NA (8.3)	NA (12.5)	NA (8.3)	NA (8.3)	NA (12.5)
Damas 2006 [26]	Cefepime + levofloxacin	By total bacteria	20	NA (17.9)	NA (3.6)	NA (7.1)	NA (10.7)	NA (3.6)	NA (14.3)	NA (0)	NA (7.1)
Heyland 2008 [27]	Meropenem + ciprofloxacin	By patient	369	31 (8.4)	11 (3.0)	47 (12.7)	19 (5.1)	30 (8.1)	30 (8.1)	6 (1.6)	16 (4.3)
Heyland 2008 [27]	Meropenem	By patient	370	16 (4.3)	4 (1.1)	52 (14.1)	23 (6.2)	31 (8.4)	39 (10.5)	8 (2.2)	6 (1.6)
Joshi 2006 [28]	Piperacillin/ tazobactam	By total bacteria	213	18 (8.5)	9 (4.2)	29 (13.6)	5 (2.3)	21 (9.9)	22 (10.3)	6 (2.8)	9 (4.2)
Joshi 2006 [28]	Imipenem/ cilastatin	By total bacteria	180	17 (9.4)	8 (4.4)	18 (10.0)	10 (5.6)	15 (8.3)	14 (7.8)	3 (1.7)	8 (4.4)
NCT00515034 [37]	Doripenem	–	–	–	–	–	–	–	–	–	–
NCT00515034 [37]	Imipenem/ cilastatin	–	–	–	–	–	–	–	–	–	–
NCT00589693 [36]	Doripenem	By qualifying gram-negative pathogens	79	17 (21.5)	15 (19.0)	–	6 (7.6)	20 (25.3)	8 (10.1)	4 (5.1)	4 (5.1)
NCT00589693 [36]	Imipenem/ cilastatin	By qualifying gram-	88	10 (11.4)	10 (11.4)	–	14 (15.9)	21 (23.9)	8 (9.1)	5 (5.7)	5 (5.7)

**Study**	**Intervention**	**Denominator**	**N**	***Pseudomonas aeruginosa*, n (%)**	***Acinetobacter baumannii*, n (%)**	***Haemophilus influenzae*, n *(%)***	***Escherichia coli*, n (%)**	***Klebsiella* spp., n (%)**	***Enterobacter* spp., n (%)**	***Proteus mirabilis*, n (%)**	***Serratia marcescens*, n (%)**
		negative pathogens									
RESTORE-IMI 1 [31]	Imipenem/ cilastatin /relebactam	–	–	–	–	–	–	–	–	–	–
RESTORE-IMI 1 [31]	Imipenem/ cilastatin + colistin	–	–	–	–	–	–	–	–	–	–
RESTORE-IMI 2 [50]	Imipenem/ cilastatin/ relebactam	–	–	–	–	–	–	–	–	–	–
RESTORE-IMI 2 [50]	Piperacillin/ tazobactam	–	–	–	–	–	–	–	–	–	–
Schmitt 2006 [32]	Piperacillin/ tazobactam	–	–	–	–	–	–	–	–	–	–
Schmitt 2006 [32]	Imipenem/ cilastatin	–	–	–	–	–	–	–	–	–	–
Torres 2000 [33]	Ciprofloxacin	By patient	41	14 (34.1)	6 (14.6)	8 (19.5)	1 (2.4)	1	3 (7.3)	0 (0)	1 (2.4)
Torres 2000 [33]	Imipenem/ cilastatin	By patient	34	12 (35.3)	3 (8.8)	13 (38.2)	1 (2.9)	1	0 (0)	1 (2.9)	0 (0)
West 2003 [35]	Levofloxacin	By patient	93	17 (18.3)	2 (2.2)	16 (17.2)	12 (12.9)	15 (16.1)	12 (12.9)	5 (5.4)	11 (11.8)
West 2003 [35]	Imipenem/ cilastatin	By patient	94	17 (18.1)	9 (9.6)	15 (16.0)	11 (11.7)	12 (12.8)	12 (12.8)	4 (4.3)	7 (7.4)
Zanetti 2003 [38]	Cefepime	By patient	77	27 (35.1)	12 (15.6)	5 (6.5)	7 (9.1)	18	4	4 (5.2)	5 (6.5)
Zanetti 2003 [38]	Imipenem/ cilastatin	By patient	71	32 (45.1)	8 (11.3)	3 (4.2)	2 (2.8)	16	4	2 (2.8)	6 (8.5)

HABP, hospital-acquired bacterial pneumonia; NA, not applicable; SLR, systematic literature review; VABP, ventilator-associated bacterial pneumonia.
