# Supplementary material for: Methods to appraise available evidence and adequacy of data from a systematic literature review to conduct a robust network meta-analysis of treatment options for patients with hospital-acquired or ventilator-associated bacterial pneumonia
Source: PLoS One. 2023 Jan 4;18(1):e0279844. doi: 10.1371/journal.pone.0279844 (PMC9812328; doi:10.1371/journal.pone.0279844)
Supplement: S5 Table — (PDF) [file pone.0279844.s008.pdf]

Methods to appraise available evidence and adequacy of data from a systematic literature review to conduct a robust network meta-analysis of treatment options for patients with hospital-acquired or ventilator-associated bacterial pneumonia

Laura Puzniak[1#], Ryan Dillon[1]*, Thomas Lodise[2]

1 Merck & Co., Inc., Rahway, New Jersey, United States of America, **2** Department of Pharmacy Practice, Albany College of Pharmacy and Health Sciences, Albany, New York, United States of America

[#]LP was an employee of Merck & Co., Inc. at the time the study was conducted

*Corresponding author

E-mail: ryan.dillon@merck.com (RD)

Short title: Network meta-analysis HABP/VABP evidence appraisal

S5 Table1. Clinical response analysis population and definition.

Study	Status at patient admission	Available populations	CE	Definition of analysis population	Definition of response
**Studies reporting clinical response (n = 4) within ASPECT-NP–connected network**					
Alvarez Lerma 2001 [22]	Empirical	ITT, CE	CE	Patients who could be assessed on the basis of clinical response, excluding cases in which the protocol was not followed, where the patient died (<72 hours after initiation of the therapy), or where there were either pathogens resistant to any of the antibiotics studied or nonbacterial pathogens	Cure: remission of the clinical manifestations of the pneumonia
Alvarez-Lerma 2001 [23]	Empirical	ITT, CE	CE	Patients with evaluable clinical response excluding protocol violations, early death (<48 hours after the initiation of treatment), isolation of pathogens resistant to some of the study drugs, and nonbacterial organisms	Cure: remission of pneumonia-related signs and symptoms
ASPECT-NP [30]	Empirical	ITT, CE	CE	Subset of the ITT population who have received study drug, adhere to the study protocol through the TOC visit, and have an evaluable clinical outcome (either cure or failure) at the TOC visit (or are classified as a clinical failure before the TOC visit)	Cure
REPROVE [34]	Empirical	cMITT, CE	CE	The CE population comprised patients in the cMITT population who received an adequate course of treatment and had an assessable clinical outcome within the assessment window, no protocol deviations that could affect the assessment of efficacy, and no	Cure: resolution of all signs and symptoms of pneumonia such that no antibacterial therapy for NP was taken between the end-of-treatment and TOC visits

				unacceptable previous or concomitant antibiotics	
**Remaining studies reporting HABP/VABP meeting SLR eligibility criteria**					
Chastre 2008 [24]	Empirical	cMITT, mMITT, CE, ME	CE	The CE population included patients who met the protocol definition of VABP, were compliant with intravenous study drug therapy, and had sufficient data available to determine clinical outcome at the TOC visit without any confounding factors that would interfere with the assessment	Cure
Chaudhary 2008 [25]	Empirical	ITT	–	–	Cure: there was disappearance of originally observed symptoms or infection
Joshi 2006 [28]	Empirical	ITT, EE	EE	To be considered EE, a patient had to (a) have a baseline pathogen susceptible to both imipenem and TZP, (b) receive the study drug for at least 5 days (or be declared a therapeutic failure after 8 doses), (c) receive no more than 1 dose of a concomitant systemic antibiotic for NP after baseline culture (unless declared a therapeutic failure), and (d) complete the TOC visit 14 (±7) days after discontinuing the study drug	Cure<br>1. Patient completed treatment, and<br>2. Did not require further antibacterial treatment to treat acute NP, and<br>3. Had improvement or lacked progression on chest radiograph, and<br>4. Showed recovery from the acute infection
NCT00515034 [37]	Empirical	CE	CE	–	Cure: complete resolution of signs and symptoms of pneumonia or lack of progression of chest X-ray abnormalities to such an extent that no further antimicrobial therapy was necessary
**NCT00589693**	**Empirical**	MITT, ME	ME	–	Clinical cure rates
RESTORE-IMI 1 [31]	Empirical	mMITT	mMITT	All randomized patients who received at least 1 dose of each trial drug within a	Sustained cure or cure

				given intravenous trial treatment regimen, and who had a baseline bacterial pathogen that met inclusion criteria	
Schmitt 2006 [32]	Confirmed	ITT	ITT	ITT population that comprised patients who had received at least 6 doses of the study medication	Response (cure/improved)
Torres 2000 [33]	Confirmed	ME	ME	Study population consisting only of patients with microbiologically confirmed NP on mechanical ventilation	Cure: signs and symptoms related to pulmonary infection had disappeared
West 2003 [35]	Empirical	ITT, CE, ME	CE	The CE population included patients with a confirmed diagnosis of NP who met study inclusion criteria and had no protocol violations during the study	Clinical success (cure or improvement)
Zanetti 2003 [38]	Empirical	PP	PP	Patients who have been treated according to the protocol (PP analysis) after exclusion of those for whom circumstances precluded classification of treatment as a success or a failure	Cure: a complete resolution of symptoms and signs and an improvement or the lack of progression of abnormalities on the chest X-ray film

CE, clinically evaluable; cMITT, clinical modified intent-to-treat; EE, efficacy evaluable; HABP, hospital-acquired bacterial pneumonia; ITT, intent-to-treat; ME, microbiologically evaluable; mMITT, microbiologic modified intent-to-treat; NP, nosocomial pneumonia; PP, per protocol; TZP, piperacillin/tazobactam; SLR, systematic literature review; TOC, test of cure; VABP, ventilator-associated bacterial pneumonia.
