# Supplementary material for: Methods to appraise available evidence and adequacy of data from a systematic literature review to conduct a robust network meta-analysis of treatment options for patients with hospital-acquired or ventilator-associated bacterial pneumonia
Source: PLoS One. 2023 Jan 4;18(1):e0279844. doi: 10.1371/journal.pone.0279844 (PMC9812328; doi:10.1371/journal.pone.0279844)
Supplement: S6 Table — (PDF) [file pone.0279844.s009.pdf]

Methods to appraise available evidence and adequacy of data from a systematic literature review to conduct a robust network meta-analysis of treatment options for patients with hospital-acquired or ventilator-associated bacterial pneumonia

Laura Puzniak[1#], Ryan Dillon[1]*, Thomas Lodise[2]

1 Merck & Co., Inc., Rahway, New Jersey, United States of America, **2** Department of Pharmacy Practice, Albany College of Pharmacy and Health Sciences, Albany, New York, United States of America

[#]LP was an employee of Merck & Co., Inc. at the time the study was conducted

*Corresponding author

E-mail: ryan.dillon@merck.com (RD)

Short title: Network meta-analysis HABP/VABP evidence appraisal

S6 Table1. All-cause mortality analysis populations and definitions.

Study	Status at patient admission	All available populations	ITT	Definition of analysis population	Time point of evaluation
**Studies reporting clinical response (n = 4) within ASPECT-NP–connected network**					
Alvarez Lerma 2001 [22]	Empirical	ITT	ITT	All patients who were randomized and who received at least 1 dose of the antibiotics proposed in the study protocol (this population was analyzed to determine the characteristics and tolerance of both treatment groups)	28 days
Alvarez-Lerma 2001 [23]	Empirical	ITT	ITT	All patients who were randomized and who received at least 1 dose of the prescribed antibiotic regimen	–
ASPECT-NP [30]	Empirical	Safety	Safety	Subset of the ITT population who receive any amount of study drug	28 days
REPROVE [34]	Empirical	Safety, cMITT, CE	Safety	All patients who received any amount of study therapy	28 days
**Remaining studies reporting HABP/VABP meeting SLR eligibility criteria**					
Ahmed 2007 [21]	Empirical	ITT	ITT	Patients who were clinically suspected to develop early onset VABP	–
Chastre 2008 [24]	Empirical	cMITT	cMITT	Safety was assessed in patients who received any study drug, that is, the ITT population	28 days
Damas 2006 [26]	Empirical	ME	ME	VABP and microbiologically confirmed	28 days
Heyland 2008 [27]	Empirical	ITT	ITT	We evaluated outcomes for all patients based on an ITT analysis	28 days
Joshi 2006 [28]	Empirical	ITT	ITT	All patients who received at least 1 dose of study drug comprised the ITT population	–

NCT00589693 [36]	Empirical	ITT, MITT	ITT	The ITT population was defined as all patients who received at least 1 dose of the study drug	28 days
RESTORE-IMI 1 [31]	Empirical	mMITT	mMITT	All randomized patients who received at least 1 dose of each trial drug within a given intravenous trial treatment regimen, and who had a baseline bacterial pathogen that met inclusion criteria	–
Schmitt 2006 [32]	Confirmed	ITT	ITT	ITT population that comprised patients who had received at least 6 doses of the study medication	–
Torres 2000 [33]	Confirmed	ME	ME	Patients who had been randomized and received at least 1 dose of the study medication	–
West 2003 [35]	Empirical	ITT	ITT	For safety data, an ITT analysis was conducted that included all enrolled patients	28–32 days
Zanetti 2003 [38]	Empirical	PP	PP	Patients who have been treated according to the protocol (PP analysis) after exclusion of those for whom circumstances precluded classification of treatment as a success or a failure	30 days

CE, clinically evaluable; cMITT, clinical modified intent-to-treat; HABP, hospital-acquired bacterial pneumonia; ITT, intent-to-treat; ME, microbiologically evaluable; mMITT, microbiologic modified intent-to-treat; PP, per protocol; SLR, systematic literature review; TOC, test of cure; VABP, ventilator-associated bacterial pneumonia.
