# Supplementary material for: Methods to appraise available evidence and adequacy of data from a systematic literature review to conduct a robust network meta-analysis of treatment options for patients with hospital-acquired or ventilator-associated bacterial pneumonia
Source: PLoS One. 2023 Jan 4;18(1):e0279844. doi: 10.1371/journal.pone.0279844 (PMC9812328; doi:10.1371/journal.pone.0279844)
Supplement: S7 Table — (PDF) [file pone.0279844.s010.pdf]

Methods to appraise available evidence and adequacy of data from a systematic literature review to conduct a robust network meta-analysis of treatment options for patients with hospital-acquired or ventilator-associated bacterial pneumonia

Laura Puzniak[1#], Ryan Dillon[1]*, Thomas Lodise[2]

1 Merck & Co., Inc., Rahway, New Jersey, United States of America, **2** Department of Pharmacy Practice, Albany College of Pharmacy and Health Sciences, Albany, New York, United States of America

[#]LP was an employee of Merck & Co., Inc. at the time the study was conducted

*Corresponding author

E-mail: ryan.dillon@merck.com (RD)

Short title: Network meta-analysis HABP/VABP evidence appraisal

S7 Table. Cochrane Central Collaboration risk-of-bias assessment part 1 of 2. All trials identified from SLR are reported below.

Study	Random sequence generation	Support for judgement	Allocation concealment	Support for judgement	Blinding of participants and personnel	Support for judgement	Blinding of outcome assessment	Support for judgement
**Studies reporting clinical response (n = 4) within ASPECT-NP–connected network**								
Alvarez Lerma 2001 [22]	Low risk	Patients were randomized by computer in a ratio of 1:1 in blocks of 6 patients, with a randomization list being generated for each hospital	Low risk	A randomization list was generated for each hospital	High risk	Open-label; participants and personnel were not blinded to treatment assignment	High risk	Open-label; outcome assessors were not blinded to treatment assignment
Alvarez-Lerma 2001 [23]	Low risk	Patients were randomized into blocks of 6 patients (4 in the study group and 2 in the control group) using a computer-generated randomization list for each hospital	Low risk	A randomization list was generated for each hospital	High risk	Open-label; participants and personnel were not blinded to treatment assignment	High risk	Open-label; outcome assessors were not blinded to treatment assignment

ASPECT-NP, Kollef 2019 [30]	Low risk	Randomization was stratified	Unclear risk	Procedures used to ensure allocation concealment was not described	Low risk	Quadruple-blind	Low risk	Quadruple-blind
REPROVE, Torres 2018 [34]	Low risk	Randomization codes were computer-generated by AstraZeneca with the AstraZeneca Global Randomization System (block size of 4)	Low risk	An interactive voice web response system was used to randomly assign patients	Low risk	Double-blind; patients, investigators, and all study center personnel were masked to study treatment	Low risk	Double-blind; patients, investigators, and all study center personnel were masked to study treatment
**Remaining studies reporting HABP/VABP meeting SLR eligibility criteria**								
Ahmed 2007 [21]	Unclear risk	Randomization procedure was not well described	Unclear risk	Procedures used to ensure allocation concealment was not described	Unclear risk	Blinding status is unclear	Low risk	Radiologists, who interpreted chest radiographs daily, were blind to the antibiotic groups
Chastre 2008 [24]	Low risk	Randomization was stratified	Unclear risk	Procedures used to ensure allocation concealment was not described	High risk	Open-label; participants and personnel were not blinded to	High risk	Open-label; outcome assessors were not blinded to

						treatment assignment		treatment assignment
Chaudhary 2008 [25]	Low risk	The randomization was done in blocks of 100 patients	Low risk	Randomization list was prepared before the start of study and random treatment assignment was placed in serially labeled, sealed envelopes	High risk	Open-label; participants and personnel were not blinded to treatment assignment	High risk	Open-label; outcome assessors were not blinded to treatment assignment
Damas 2006 [26]	Unclear risk	Randomization procedure was not well described	Unclear risk	Procedures used to ensure allocation concealment was not described	High risk	Open-label; participants and personnel were not blinded to treatment assignment	High risk	Open-label; outcome assessors were not blinded to treatment assignment
Heyland 2008 [27]	Low risk	Randomization was stratified	Low risk	Randomized was done using a central telephone system	High risk	Open-label; participants and personnel were not blinded to treatment assignment	High risk	Open-label; outcome assessors were not blinded to treatment assignment
Joshi 2006 [28]	Low risk	A computer-generated randomization	Unclear risk	Procedures used to ensure allocation	Low risk	Double-blind	Low risk	Double-blind

		schedule assigned patients in a 1:1 ratio		concealment was not described				
NCT00515034 [37]	Unclear risk	Randomization procedure was not well described	Unclear risk	Procedures used to ensure allocation concealment was not described	High risk	Open-label; participants and personnel were not blinded to treatment assignment	High risk	Open-label; outcome assessors were not blinded to treatment assignment
NCT00589693 [36]	Unclear risk	Randomization procedure was not well described	Unclear risk	Procedures used to ensure allocation concealment was not described	Low risk	Double-blind, double-dummy	Low risk	Double-blind, double-dummy
RESTORE-IMI 1 [31]	Low risk	Randomization was stratified	Unclear risk	Procedures used to ensure allocation concealment was not described	Low risk	Double-blind	Low risk	Double-blind
RESTORE-IMI 2 [50]	–	–	–	–	Low risk	Double-blind	Low risk	Double-blind
Schmitt 2006 [32]	Low risk	Randomization schedule unknown to the investigator was used	Low risk	The investigator was not informed about the treatment assigned to any patient or	Low risk	Double-blind	Low risk	Double-blind

				about the randomization code				
Torres 2000 [33]	Unclear risk	Randomization procedure was not well described	Low risk	Closed envelopes were distributed to each center before the study to ensure randomization	High risk	Open-label; participants and personnel were not blinded to treatment assignment	High risk	Open-label; outcome assessors were not blinded to treatment assignment
West 2003 [35]	Low risk	Patients were randomly assigned to treatment groups using a computer-generated centralized randomization schedule	Low risk	Randomization was centralized	High risk	Open-label; participants and personnel were not blinded to treatment assignment	High risk	Open-label; outcome assessors were not blinded to treatment assignment
Zanetti 2003 [38]	Unclear risk	Randomization procedure was not well described	Low risk	Centralized telephone randomization system	High risk	Open-label; participants and personnel were not blinded to treatment assignment	Low risk	Evaluator-blind

HABP, hospital-acquired bacterial pneumonia; SLR, systematic literature review; VABP, ventilator-associated bacterial pneumonia.
