# Supplementary material for: Methods to appraise available evidence and adequacy of data from a systematic literature review to conduct a robust network meta-analysis of treatment options for patients with hospital-acquired or ventilator-associated bacterial pneumonia
Source: PLoS One. 2023 Jan 4;18(1):e0279844. doi: 10.1371/journal.pone.0279844 (PMC9812328; doi:10.1371/journal.pone.0279844)
Supplement: S8 Table — (PDF) [file pone.0279844.s011.pdf]

Methods to appraise available evidence and adequacy of data from a systematic literature review to conduct a robust network meta-analysis of treatment options for patients with hospital-acquired or ventilator-associated bacterial pneumonia

Laura Puzniak[1#], Ryan Dillon[1*], Thomas Lodise[2]

1 Merck & Co., Inc., Rahway, New Jersey, United States of America, **2** Department of Pharmacy Practice, Albany College of Pharmacy and Health Sciences, Albany, New York, United States of America

[#]LP was an employee of Merck & Co., Inc. at the time the study was conducted

*Corresponding author

E-mail: ryan.dillon@merck.com (RD)

Short title: Network meta-analysis HABP/VABP evidence appraisal

S8 Table. Cochrane Central Collaboration risk-of-bias assessment part 2 of 2. All trials identified from SLR are reported below.

Study	Incomplete outcome data	Support for judgement	Selective reporting	Support for judgement	Other sources of bias	Support for judgement
**Studies reporting clinical response (n = 4) within ASPECT-NP–connected network**						
Alvarez Lerma 2001 [22]	Low risk	Patient ineligibility and discontinuation were explained in the "Results" section. Missing outcome data balanced in numbers across intervention groups, with similar reasons for missing data across groups	Low risk	The study protocol is not available, but it is reasonably clear that the published reports include all expected outcomes	Low risk	No indication there are other sources of bias
Alvarez-Lerma 2001 [23]	Low risk	Patient ineligibility and discontinuation were explained in Table 1. Missing outcome data balanced in numbers across intervention groups, with similar reasons for missing data across groups	Low risk	The study protocol is not available, but it is reasonably clear that the published reports include all expected outcomes	Low risk	No indication there are other sources of bias
ASPECT-NP [30]	Low risk	Patient ineligibility and discontinuation were explained in the "Participant flow" table. Missing outcome data balanced in numbers	Low risk	The study protocol is not available, but it is reasonably clear that the published reports include all expected outcomes	Low risk	No indication there are other sources of bias

Study	Incomplete outcome data	Support for judgement	Selective reporting	Support for judgement	Other sources of bias	Support for judgement
		across intervention groups, with similar reasons for missing data across groups				
REPROVE [34]	Low risk	Patient ineligibility and discontinuation were explained in Fig 1. Missing outcome data balanced in numbers across intervention groups, with similar reasons for missing data across groups	Low risk	The study protocol is not available, but it is reasonably clear that the published reports include all expected outcomes	Low risk	No indication there are other sources of bias
**Remaining studies reporting HABP/VABP meeting SLR eligibility criteria**						
Ahmed 2007 [21]	Low risk	Patient ineligibility and discontinuation were explained in Table 2. Missing outcome data balanced in numbers across intervention groups, with similar reasons for missing data across groups	Low risk	The study protocol is not available, but it is reasonably clear that the published reports include all expected outcomes	Low risk	No indication there are other sources of bias
Chastre 2008 [24]	Low risk	Patient ineligibility and discontinuation were explained in Table 1. Missing outcome data balanced in numbers across intervention groups, with similar	Low risk	The study protocol is not available, but it is reasonably clear that the published reports include all expected outcomes	Low risk	No indication there are other sources of bias

Study	Incomplete outcome data	Support for judgement	Selective reporting	Support for judgement	Other sources of bias	Support for judgement
		reasons for missing data across groups				
Chaudhary 2008 [25]	Low risk	Patient ineligibility and discontinuation were explained in Fig 1. Missing outcome data balanced in numbers across intervention groups, with similar reasons for missing data across groups	Low risk	The study protocol is not available, but it is reasonably clear that the published reports include all expected outcomes	Low risk	No indication there are other sources of bias
Damas 2006 [26]	Low risk	Patient ineligibility and discontinuation were explained in the "Results" section. Missing outcome data balanced in numbers across intervention groups, with similar reasons for missing data across groups	Low risk	The study protocol is not available but it is reasonably clear that the published reports include all expected outcomes	Low risk	No indication there are other sources of bias
Heyland 2008 [27]	Low risk	Patient ineligibility and discontinuation were explained in Fig 1. Missing outcome data balanced in numbers across intervention groups, with similar reasons for missing data across groups	Low risk	The study protocol is not available, but it is reasonably clear that the published reports include all expected outcomes	Low risk	No indication there are other sources of bias

Study	Incomplete outcome data	Support for judgement	Selective reporting	Support for judgement	Other sources of bias	Support for judgement
Joshi 2006 [28]	Low risk	Patient ineligibility and discontinuation were explained in Fig 1. Missing outcome data balanced in numbers across intervention groups, with similar reasons for missing data across groups	Low risk	The study protocol is not available, but it is reasonably clear that the published reports include all expected outcomes	Low risk	Drs Joshi, Metzler, McCarthy, Olvey, and Kassira received research funding from the study sponsor, Wyeth Research. Ms Cooper is an employee of Wyeth Research
NCT00515034 [37]	Low risk	Patient ineligibility and discontinuation were explained in the "Participant flow" table. Missing outcome data balanced in numbers across intervention groups, with similar reasons for missing data across groups	Low risk	The study protocol is not available, but it is reasonably clear that the published reports include all expected outcomes	Low risk	No indication there are other sources of bias
NCT00589693 [36]	Unclear risk	Patient ineligibility and discontinuation were not described	Low risk	The study protocol is not available, but it is reasonably clear that the published reports include all expected outcomes	High risk	The study was terminated early based on recommendation from an independent data monitoring committee
RESTORE-IMI 1 [31]	Low risk	Patient ineligibility and discontinuation were explained in the	Low risk	The study protocol is not available, but it is reasonably	Low risk	No indication there are other sources of bias

Study	Incomplete outcome data	Support for judgement	Selective reporting	Support for judgement	Other sources of bias	Support for judgement
		"Participant flow" table. Missing outcome data balanced in numbers across intervention groups, with similar reasons for missing data across groups		clear that the published reports include all expected outcomes		
RESTORE-IMI 2 [50]	–	–	–	–	–	–
Schmitt 2006 [32]	Low risk	Patient ineligibility and discontinuation were explained in Fig 1. Missing outcome data balanced in numbers across intervention groups, with similar reasons for missing data across groups	Low risk	The study protocol is not available, but it is reasonably clear that the published reports include all expected outcomes	Low risk	No indication there are other sources of bias
Torres 2000 [33]	Low risk	Patient ineligibility and discontinuation were explained in Fig 1. Missing outcome data balanced in numbers across intervention groups, with similar reasons for missing data across groups	Low risk	The study protocol is not available, but it is reasonably clear that the published reports include all expected outcomes	Low risk	No indication there are other sources of bias
West 2003 [35]	Low risk	Patient ineligibility and discontinuation were	Low risk	The study protocol is not available, but	Low risk	No indication there are other sources of bias

Study	Incomplete outcome data	Support for judgement	Selective reporting	Support for judgement	Other sources of bias	Support for judgement
		explained in Table 2. Missing outcome data balanced in numbers across intervention groups, with similar reasons for missing data across groups		it is reasonably clear that the published reports include all expected outcomes		
Zanetti 2003 [38]	Low risk	Patient ineligibility and discontinuation were explained in the "Results" section. Missing outcome data balanced in numbers across intervention groups, with similar reasons for missing data across groups	Low risk	The study protocol is not available, but it is reasonably clear that the published reports include all expected outcomes	Low risk	No indication there are other sources of bias

HABP, hospital-acquired bacterial pneumonia; SLR, systematic literature review; VABP, ventilator-associated bacterial pneumonia.
